# Erosive Hand Osteoarthritis is Associated with Subclinical Atherosclerosis and Endothelial Dysfunction

**Published:** 2013-12

**Authors:** Athanasios Koutroumpas, Athanasios Giannoukas, Elias Zintzaras, Ekaterini Exarchou, Aris Baliakos, Konstantinos Makaritsis, Lazaros I. Sakkas

**Affiliations:** 1 Department of Rheumatology, Faculty of Medicine, School of Health Sciences, University of Thessaly, Greece;; 2 Department of Vascular Surgery, Faculty of Medicine, School of Health Sciences, University of Thessaly, Greece;; 3 Department of Biomathematics, Faculty of Medicine, School of Health Sciences, University of Thessaly, Greece;; 4 Center for Molecular Medicine, Old Dominion University, Norfolk, VA, USA

**Keywords:** atherosclerosis, Doppler ultrasonography, dilatation, endothelium, erosive osteoarthritis, Framingham risk score

## Abstract

Chronic inflammatory disorders have been associated with accelerated atherosclerosis and increased cardiovascular (CV) risk. Recent evidence suggests that erosive hand osteoarthritis (EOA) has considerable inflammation; therefore, we examined the presence of subclinical atherosclerosis and endothelial dysfunction in EOA. Twenty-four patients with EOA and 24 age- and sex-matched healthy individuals without clinical OA were included in the study. No subject had a history of CV disease. Intima-media thickness (IMT) and atheromatous plaques in the common carotid and common femoral arteries were measured by Doppler ultrasonography. The endothelium-dependent, flow-mediated dilatation (FMD) and endothelium-independent, sublingual glyceryl trinitrate (NTG)-induced dilatation (NMD) of the brachial artery were assessed. The EOA patients had significantly elevated systolic and diastolic blood pressure (*p*<0.001 for both). The 10-year risk of general CV disease, as predicted with the Framingham Risk Score, was similar in patients and controls (*p*=0.18). IMT of both common carotid and common femoral artery were increased in EOA (*p*=0.01 and *p*<0.01, respectively), but the frequency of atherosclerotic plaques was not increased. There was no difference in FMD and NMD between the two groups, but the difference between FMD and NMD was increased in EOA. In conclusion, this small controlled study showed an association between EOA and subclinical atherosclerosis that cannot be fully attributed to traditional CV risk factors, as assessed by the Framingham score. These results suggest that chronic, low-grade inflammation is implicated in atherosclerosis in EOA.

## INTRODUCTION

Erosive hand osteoarthritis (EOA) is a subset of hand osteoarthritis (HOA) which is defined by erosions in interphalangeal (IP) joints on plain radiographs and occurs primarily in postmenopausal women (1). Traditionally, osteoarthritis (OA) has been considered a degenerative disorder of the aging population. However, synovial inflammation has been frequently described in early and advanced knee or hip OA (2, 3) and inflammation is increasingly recognized as an important factor in the pathophysiology of this disease (4, 5). Newer imaging modalities, such as ultrasound and MRI, revealed frequent local inflammation in HOA (6, 7). The inflammatory component is more evident in EOA (1, 8, 9). Systemic low-grade inflammation, as exemplified by elevated serum C-reactive protein, has been reported in EOA (10).

A growing body of evidence supports that endothelial dysfunction and atherosclerosis are significantly more prevalent in patients with chronic inflammatory disorders, compared to age-matched controls (11). This has been convincingly demonstrated in rheumatoid arthritis, a prototype of chronic inflammatory diseases, which is associated with increased cardiovascular (CV) risk (12). Therefore, we sought to investigate the occurrence of atherosclerosis and endothelial dysfunction in patients with EOA compared to normal controls.

## METHODS

### Selection and description of participants

Patients participating in the study were followed at the Rheumatology outpatient clinic of the University General Hospital of Larissa. All fulfilled the 1990 American College of Rheumatology criteria for the classification of HOA (13).  In addition, they exhibited IP joint central area erosions in the form of “gull-wing” or “saw-teeth” on plain radiography and classified as having EOA. A second group of age- and sex-matched individuals served as healthy controls, with no hand pain or clinical signs of hand osteoarthritis, such as Heberden’s and Bouchard’s nodes, or 1^st^ carpometacarpal joint squaring. In addition, they had no history or clinical signs and symptoms of osteoarthritis in other joints. All participants, in both groups, had no history of CV disease, such as an acute coronary syndrome, stroke or peripheral arterial disease. History of diabetes mellitus (DM), hypertension (HT), hyperlipidemia (HL) and smoking, and any relevant medication were recorded.

The study protocol was approved by the Institutional Scientific and Ethical Committee, all participants gave a written informed consent, and the study was conducted according to good clinical practice.

### Technical information

Ultrasonographic (US) examination was performed by a single trained and senior vascular surgeon (GA). Common carotid and common femoral artery intima-media thickness (IMT), were measured using high resolution B-mode ultrasonography, using a 12 L, 5 Hz linear transducer (GE Logic equipment), according to recently published recommendations (14). Measurements were conducted twice for both the right and the left artery, and the mean values were recorded. IMT was used as a marker of preclinical atherosclerosis, as common carotid artery IMT is an early atherosclerotic index and a strong predictor of cardiovascular end-points, such as stroke and coronary events (15, 16).

The presence and type of atheromatous plaques was recorded for both sites. A plaque was defined as a focal structure encroaching into the lumen, of at least 0.5 mm, or 50% of the surrounding IMT. Alternatively, a plaque should demonstrate thickness >1.5 mm, measured from the media-adventitia to the media-lumen interface (14). Carotid plaques were characterized as type 1-5, depending on the echogenicity and homogeneity of the plaque (17). This characterization has clinical implications, as type 1 and 2 plaques carry a high risk for complications, type 3 and 4 plaques carry a low risk, and type 5 calcified plaques carry an undetermined risk (18).

Two definitions were used for subclinical atherosclerosis: plaque formation and a common carotid artery IMT >1 mm. The cut-off of 1 mm was used because this level is associated with coronary artery disease and increased CV risk (18). As common carotid artery IMT is a better surrogate for CV risk than common femoral artery IMT, risk analyses were performed using carotid artery measurements.

An US “biopsy score” (USBS) has been assessed in all subjects (19). This is a non-invasive procedure for the detection and grading of early carotid and femoral artery atherosclerosis. Subjects were graded into A-F classes, according to vessel wall characteristics: (a) normal (intima media and adventitia clearly separated), 0 points; (b) interface disruption, 2 points; (c) intima-media granulation, 4 points; (d) plaque without hemodynamic disturbance, 6 points; (e) asymptomatic plaque causing stenosis, 8 points; (f) plaque causing stenosis and symptoms, 10 points. The points for the two carotid and two femoral arteries were added, giving a numerical score ranging from 0 to 40. Classes D-E are associated with higher risk of ischemic cardiovascular or cerebrovascular events and higher risk of mortality than classes A-C (20).

Endothelial function was assessed by measuring flow-mediated endothelium-dependent dilatation (FMD). The end-diastolic diameter of both brachial arteries was measured at rest and within 15 sec after release of a sphygmomanometer cuff inflated at a pressure of at least 50 mmHg above the subject’s systolic blood pressure (BP) for 3 minutes (21). Similar measurements were conducted 5 minutes after administration of 0.4 mg of glyceryl trinitrate (NTG) sublingually for the assessment of endothelium-independent dilatation (NMD) (21). Vasodilatation was expressed as the difference between the two measurements. FMD reflects endothelial dysfunction and is mediated by the release of nitric oxide from arterial endothelial cells. It is predictive of CV events, and is related to both the presence and extent of coronary atherosclerosis (22).

As the vasodilator potential is not the same across individuals, NMD can be used as a means of measuring the maximum obtainable vascular wall reaction to a vasodilatory stimulus (23). Therefore, we calculated the difference between NMD and FMD and compared the means between the two groups of individuals. In this way, FMD is opposed not just to the baseline brachial artery diameter, but also to the maximum dilatation obtained by administration of NTG. Apart from baseline diameter, dilatation is influenced by hypertension and vasoactive anti-hypertensive drugs. All differences were expressed as numerical values (in mm), rather than percentage of change from baseline value. Expressing the vasodilator effect as the percentage of the baseline value is more suitable for measurements in the same individual before and after an intervention (23).

All assessments were conducted in a quiet, dark room and subjects were examined in the supine position. Patients remained at the supine position for at least 10 minutes before the examination.

Serum total cholesterol, high density lipoprotein (HDL), low density lipoprotein (LDL) and triglycerides were measured by standard methods. The cut-off values for total cholesterol, LDL and HDL are in accordance with the Third Report of the National Cholesterol Education Program (NCEP) (24).

The Framingam 10-year risk of CVD was estimated according to published method (25). This algorithm incorporates age, sex, systolic blood pressure, smoking, blood pressure treatment and LDL and HDL levels and provides an estimate of the 10-year risk of clinical CVD. The Framingham risk score provides the risk that is attributed to traditional CVD risk factors.

### Statistics

Qualitative variables were compared with the Fischer’s exact test and the chi-square test, as appropriate. Comparisons between means were carried out with the Student’s t-test and the Mann-Whitney U, as appropriate. Statistical analysis has been performed using the SPSS, version 13, software (SPSS Inc. Chicago, USA).

For each group the risk of preclinical atherosclerosis was separately assessed with regard to age, sex, history of hypertension, hyperlipidaemia, diabetes and smoking, total cholesterol (whether >240 or not), LDL (whether >160 or not), HDL (whether <50 or not), systolic blood pressure (whether > or <135 mmHg, which is the upper normal limit, according to international guidelines) and diastolic blood pressure (whether > or <90 mmHg), and regular use of vasoactive and hypolipidaemic agents (β-blockers, calcium channel blockers, angiotensin converting enzyme inhibitors, angiotensin II receptor antagonists, diuretics and statins), with the Fisher’s exact test and the Mann-Whitney U test.

## RESULTS

### Demographics and lipids

The two groups were comparable for all demographic variables. No significant difference was found in the frequency of diabetes mellitus, hypertension, hyperlipidaemia and smoking status between EOA patients and controls. Similarly, no difference was found in the rate of prescription of vasoactive and hypolipidemic medications between the two groups (Table 1). Fourteen of the EOA patients (58%) were receiving hydroxychloroquine (HCQ).

**Table 1:** Demographic characteristics of patients with EOA and normal controls

Parameter	EOA (n=24)	Controls (n=24)	*p*

Age, mean (SD)	62.5 (6.6)	60.7 (5.8)	0.33
Sex (Female)	22	22	0.99
History-Hypertension, n (%)	16 (66.6)	12 (50)	0.38
Hyperlipidemia, n (%)	7 (29.2)	13 (54.2)	0.14
Diabetes mellitus, n (%)	0 (0)	2 (8.3)	0.49
Smoking, n (%)	3 (12.5)	4 (16.7)	0.99
Treatment-statin, n (%)	4 (16.7)	8 (33.3)	0.32
ACE inhibitor, n (%)	6 (25)	3 (12.5)	0.46
Beta blocker, n (%)	2 (8.3)	6 (25)	0.244
AT II receptor blockers, n (%)	5 (20.8)	3 (12.5)	0.46
Diuretics, n (%)	6 (25)	5 (20.8)	0.74
Hydroxychloroquine, n (%)	14 (58.3)	0 (0)	<0.0001

SD, standard deviation; ACE, angiotensin converting enzyme; AT II, Angiotensin II; NS, non-significant.

Serum lipid concentrations were available for 23 of the 24 subjects in the EOA group and all subjects in the control group. There was a non-significant trend towards increased serum lipid concentrations for total cholesterol, LDL and triglycerides in the EOA group (Table 2). EOA patients had significantly elevated systolic (160 ± 32.9 mmHg [mean ± SD]) and diastolic (99.9 ± 20.4 mmHg) blood pressure (BP) at the time of examination, compared to the controls (systolic BP = 143.4 ± 32.1 mmHg, diastolic BP = 83.42 ± 11.9 mmHg) (*p*<0.001 for both) (Table 2).

**Table 2:** Serum lipid profiles and blood pressure measurements in patients with EOA and normal controls

Variable	EOA (n=24)	Controls (n=24)	*P*

Cholesterol (mg/dL), mean (SD)	237.6 (49.5)	230 (35.4)	0.55
HDL (mg/dL), mean (SD)	59.4 (14.3)	59.1 (13.1)	0.94
LDL (mg/dL), mean (SD)	157.5 (48.8)	147 (33.3)	0.37
Triglycerides (mg/dL), mean (SD)	141.8 (75.2)	124.4 (56)	0.63
Systolic BP (mmHg), mean (SD)	160 (32.9)	143.4 (32.1)	<0.001
Diastolic BP (mmHg), mean (SD)	99.9 (20.4)	83.42 (11.9)	<0.001

The Framingam 10-year risk of CVD was calculated for 23 of the 24 participants in the EOA group, as 1 patient lacked serum lipid determinations. Patients with EOA had a non-significant trend towards an increased Framingham risk score (mean=23.9% 10-year risk of CVD), compared to the control group (mean=17.3% 10-year risk of CVD, *p*=0.18) (Table 3).

**Table 3:** Intima media thickness and flow-mediated and nitroglycerine-mediated vasodilatation in patients with EOA and normal controls

Variable	EOA (n=24)	Controls (n=24)	*P*

Framingham risk score (10-year CVD rate, mean, SD)	23.9 (14.7)	18.8 (11.2)	0.18
Intima-media thickness, common carotid artery (mm), mean (SD)	0.91 (0.17)	0.82(0.19)	0.012
Intima-media thickness, common femoral artery (mm), mean (SD)	0.7 (0.19)	0.6 (0.2)	<0.01
Ultrasound Biopsy Score, mean (SD)	14 (3.3)	12 (2.8)	0.03
Ultrasound Biopsy Score, no of patients with at least one Class D-F plaque (%)	15 (62.5)	13 (54.2)	0.77
Δ Brachial artery diameter- reactive hyperemia (flow-mediated vasodilatation-FMD) (mm), mean (SD)	0.36 (0.39)	0.36 (0.44)	0.92
Δ Brachial artery diameter after glyceryl Trinitrate administration (NMD) (mm), mean (SD)	0.62 (0.49)	0.45 (0.48)	0.057
Δ Brachial artery diamener FMD-NMD	0.348 (0.2)	0.1 (0.49)	0.026

### Ultrasonography

EOA was associated with a marginally significant increase in the risk of IMT>1mm (OR=3.33, 95% CI 1.02-10.9, *p*=0.043) (Table 4). Common carotid artery IMT was significantly elevated in the EOA group, compared to the control group (*p*=0.012). Similarly, common femoral artery IMT was elevated in the EOA compared to the control group (*p*<0.01) (Table 3). Fifteen EOA patients (62.5%) had carotid and/or femoral artery atherosclerotic plaques, identified by high-resolution B-mode ultrasonography, compared to 13 controls (54.2%) with atherosclerotic plaques, but this difference was not statistically significant (*p*=0.77). For each type of plaque, the difference between the two groups was not statistically significant (data not shown). One patient in the EOA group had occluded internal carotid artery. All other plaques were not hemodynamically significant. EOA was not associated with an increased risk of plaque, compared to controls.

**Table 4:** Risk of subclinical atherosclerosis in EOA patients and normal controls

Lesion	EOA (n=24)	Controls (n=24)	OR

IMT>1mm, n (%)	16 (66.7)	9 (37.5)	OR=3.33 (95% CI 1.02-10.9)
			*p*=0.043 (chi-square test)
			*P*=0.08 (Fischer’s exact test)
Plaque, n (%)	15 (62.5)	13 (54.2)	OR=1.41 (95% CI 0.46-4.46)
			*P*=0.77

The total USBS was higher in the EOA group compared to the controls (*p*=0.03) (Table 3). As classes D-F are associated with higher risk of ischemic events, we compared the number of subjects in each group that had at least one class D-F plaque. A non-significant trend in favor of the EOA group was found (Table 3). For each group the risk of subclinical atherosclerosis did not correlate with any demographic characteristic, lipids, blood pressure, or medications, thus no effect modifiers were found. Therefore, no multivariate analysis was required.

### Flow-mediated and nitroglycerine induced vasodilatation

Twenty-two individuals in both groups received glyceryl trinitrate for the assessment of endothelium-independent vasodilatation. Four individuals did not receive glyceryl trinitrate because of aortic valve stenosis (one individual) and hypotension (3 individuals). No difference was found between the two groups in both flow-mediated, endothelium-dependent vasodilatation and nitroglycerine-induced, endothelium-independent vasodilatation. However, the difference between FMD and NMD was significantly greater in EOA patients compared to healthy controls (*p*=0.026) (Table 3). Neither NMD nor FMD was correlated with systolic or diastolic BP.

## DISCUSSION

In this controlled study we found evidence of subclinical atherosclerosis and endothelial dysfunction in EOA. This is in agreement with a recent Rotterdam population-based study in persons aged >55 years that investigated carotid atherosclerosis and found an association with distal IP OA in women (26). Similarly, a population-based study in Iceland found hand OA in elderly women to be associated with carotid and coronary atherosclerosis (27). In the Framingham Heart study, an association was found between spinal facet joint OA and abdominal aortic calcifications, a surrogate for CV disease, after adjustment for epidemiologic factors associated with traditional CV risk factors (28). Also, patients with generalized OA had significantly increased popliteal artery wall thickness, assessed by MRI (29). However, other studies have not found association of hand OA with arterial stiffness (30) or abdominal aortic calcification (31).

Previous studies showed increased incidence of CV events and increased mortality in patients with OA compared to the general population (32-34). The enhanced CV risk in OA could be, at least in part, explained by the high prevalence of co-morbidities and traditional CV risk factors among older subjects with OA. Indeed, in the large population of the third National Health and Nutrition Examination Survey, 40% of the OA patients had hypertension, 11% had diabetes and 32% had high total cholesterol, compared with 25%, 6% and 24%, respectively, of the US general population without arthritis (35). The Ulm study, in OA patients undergoing hip or knee surgery, showed a positive correlation of hypercholesterolaemia with widespread joint involvement (36). In our study, EOA did not exhibit a significant increase in the 10-year risk of CVD, as predicted by the Framingham Risk Score, which is based on the contribution of traditional CV risk factors.

We found an increased intima-media thickness (subclinical atherosclerosis) in patients with EOA that could not be attributed to traditional CV risk factors, as assessed by the 10-year Framingham risk score. Moreover, patients with EOA exhibited evidence of endothelial dysfunction, as estimated by the difference between FMD and NMD, that could not be attributed to differences in systolic or diastolic blood pressure.

The link between OA and atherosclerosis could be explained by shared pathogenetic mechanisms. Inflammation appears to play a role in OA and EOA in particular (1, 5, 8-10). Chronic inflammation also promotes endothelial cell activation and endothelial dysfunction (11). Inflammatory mediators, such as tumor necrosis factor-α (TNF-α), interleukin (IL)-6 and IL-1, up-regulated in OA (5), participate in the pathophysiology of atherosclerosis (37, 38). C-reactive protein, elevated in EOA (10), is associated with atherosclerosis and is predictive of future ischemic events (39, 40). Osteoprotegerin (OPG), part of the Receptor Activator Nuclear Factor κ-B (RANK)/RANK ligand system, inhibits osteoclastogenesis, and thus bone resorption, is up-regulated in hand OA (41). Elevated serum OPG levels have been associated with coronary artery disease and stroke and have been proposed as an independent risk factor for CV disease and associated mortality (42). Adiponectin may be a link between EOA and atherosclerosis. High adiponectin levels are associated with a decreased risk of radiographic progression in hand OA (43). Low levels of adiponectin are associated with obesity and CV disease. Adiponectin reduces lipid accumulation in macrophage foam cells. Macrophages transduced with adiponectin gene exhibit decreased uptake of oxidized low-density lipoprotein and increased HDL-mediated cholesterol efflux (44). However, the precise molecular basis of both disorders is still evading us.

A limitation of our study is the small sample size, and therefore, our results should be confirmed in a larger study. However, US examinations were performed by a single, experienced assessor, ruling out inter-observer variations. In addition, 14 out of 24 EOA patients (58.3%) were receiving HCQ for a mean period of 23.5 months. HCQ might have improved vascular function in our study population, since it has been shown to exert a mild protective effect against atherosclerosis in patients with systemic lupus erythematosus (45). This means that the difference between the two populations would have been more significant, in the absence of HCQ.

In conclusion, in this small, cross-sectional, controlled study, we found evidence of subclinical atherosclerosis and endothelial dysfunction in patients with EOA.
